# Quality Improvement Initiative to Improve Hand Hygiene Compliance in Indian Special Newborn Care Unit

**DOI:** 10.1097/pq9.0000000000000492

**Published:** 2021-12-15

**Authors:** Reena Rai, Amanpreet Sethi, Amarpreet Kaur, Gurmeet Kaur, Harsh Vardhan Gupta, Sumandeep Kaur, Man Singh Parihar, Satwinder Paul Singh

**Affiliations:** From the * Department of Pediatrics, Guru Gobind Singh Medical College and Hospital, Faridkot, Punjab, India; † Department of Pediatric Nursing, University College of Nursing, Faridkot, Punjab, India.

## Abstract

Pilot data on Hand hygiene (HH) compliance using a standard World Health Organisation checklist for 1-week suggested only 20% compliance. So, we planned a Quality Improvement study to improve HH compliance among health care providers in our Special Newborn Care Unit from 20% to 60% over 12 months.

**Methods:**

We did this study in 3 phases: Baseline phase (2 months), Intervention phase (8 months), and Postintervention phase (2 months). A multidisciplinary Quality Improvement team composed of doctors, nursing staff, and ward attendants was constituted. The team analyzed potential barriers to HH by Fishbone analysis. Three trained observers randomly selected two target Special Newborn Care Unit patients daily and collected data on HH compliance unobtrusively during the three 8-h shifts over 24 h. In addition, we tested a range of interventions using multiple Plan Do Study Act cycles: Staff education; Displaying posters; Round the clock availability of soap and hand rub; Staff felicitation; Group performance feedback. We also collected data on healthcare-associated infections in all three phases.

**Results:**

The total observations for HH during the baseline, intervention, and postintervention phase were 1488, 5808, and 1464, respectively. The HH compliance improved from 27.2% to 57.1% in the postintervention phase. There was no difference in the healthcare-associated infections among the three phases.

**Conclusions:**

The HH compliance rates improved significantly but not to the desired extent. So, we planned to increase our workforce, and improve our training program and infrastructure.

## Abstract 2

Supplemental Digital Content is available in the text.

## INTRODUCTION

Neonatal sepsis is the second most common cause of mortality after prematurity in neonates in India, accounting for 9.9 deaths per 1000 live births.^1^ India Newborn Action Plan launched in 2014 an initiative to reduce the neonatal mortality rate to less than 10 per 1000 live births. One of the most critical intervention packages is to improve the care of small and sick newborns in Special Newborn Care Units (SNCU) by addressing the issues like lack of adequate and skilled nursing staff, lack of functional equipment, and overcrowding. Also, we must improve poor infection control practices like low hand hygiene (HH) compliance, poor follow-up care postdischarge, and increased out-of-pocket expenses.^2^ Small and sick newborns are most susceptible to infections because of their immature host defense mechanisms. Moreover, frequent use of antibiotics and invasive interventions in the neonatal intensive care unit (NICU) often puts them at a greater risk. Organisms responsible for nosocomial infections in NICUs are often transmitted by doctors, nursing, and allied staff.^3^ So, meticulous HH by all healthcare providers (HCPs) is one of the most critical infection control practices that can prevent Health Care-Associated Infection (HCAI).

Poor adherence to HH compliance is widespread, especially in ICU settings. In a worldwide systematic review on HH compliance comprising 96 studies, Erasmus et al revealed that the rate of HH compliance among all health care workers according to HH guidelines was only 30%–40%, especially in an intensive care setting.^4^ A recent observational study in the hospitals of two states from Southern India (Telangana and Andhra Pradesh) also demonstrated overall HH compliance rates of 23% in newborn care units, with public health facilities showing a compliance rate of only 12%.^5^ A recent Quality Improvement (QI) study by Kallam et al, in 2018, done in Ghana, showed improvement in HH compliance in the NICU from 67% to 92% using the WHO-recommended Multimodal change package, which included the creation of a training course on hand hygiene, re-enforcement of HH practices among staff, visual reminders of HH practices, and round-the-clock availability of clean hand towels for drying.^6^

Over the last 18 months, we retrospectively observed that the proportion of sepsis (both culture-positive and culture-negative clinical sepsis) among all the admitted neonates in our SNCU was 39.5%, a very high rate compared with the national average of 18%.^7^ The mortality attributed to sepsis was almost 31%, which is also high compared with the national average of 15%.^7^ In the next step, we collected pilot data on HH compliance using the WHO checklist for 1 week and found that compliance was only 20%. So, we planned a multimodal intervention study using QI methodology to improve HH compliance from 20% to 60% in SNCU over 12 months to decrease the incidence of HCAIs among admitted neonates. We assumed that by doing this QI project, we would be able to infuse the characteristics of teamwork, accountability, and patient safety among health care staff, thus improving the quality of care.

## METHODS

### Setting

The study unit is a tertiary-level 12 bed SNCU and regional referral center in Punjab, India. We cater to sick neonates (inborn, out-born) requiring mechanical ventilation and neonates with surgical conditions. There is no separate out-born and step-down unit. The average annual admission rate is 1000 per year. The proportion of admitted neonates according to birth weight is less than 1000 g: 3.6%; 1000–1500 g: 15.4%; and 1500–2500: 50.4%. The unit has three resident doctors, two staff nurses, and one ward attendant on each shift. There are two interconnected rooms and only one laboratory sink for handwashing, with no separate hand sanitizer stations and displayed HH policy on display at the start of the project.

### Study Period

The timeline of the project was from July 1, 2018 to June 30, 2019 (12 months). The study subjects were the caregivers (nurses, doctors, and allied health personnel) working in the SNCU.

**Fig. 1.**
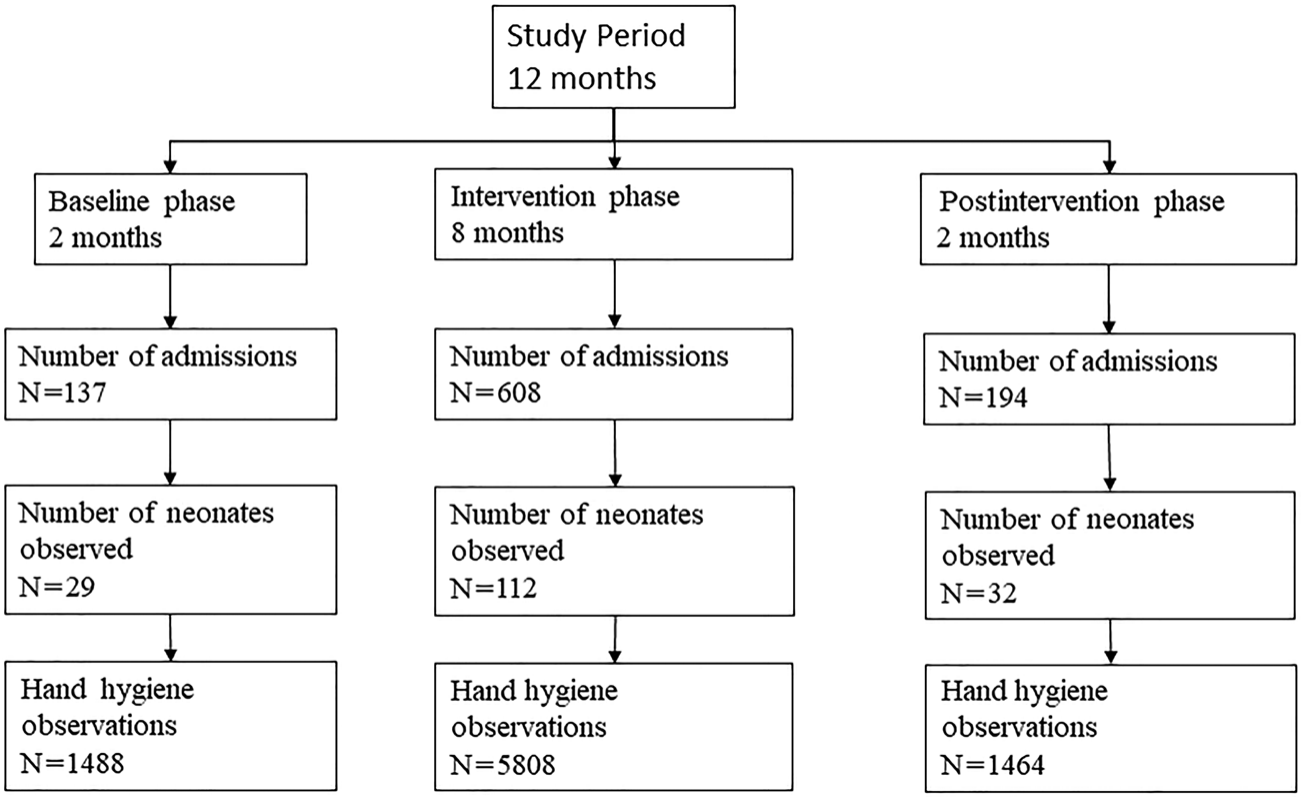
Study Flow.

After the Institute’s ethical committee approval, the QI project was initiated. We explained to the HCPs the purpose of the study, and strict confidentiality was ensured. Written informed consent was taken from respective caregivers before the study data collection. The project was completed in three phases: Baseline phase-2 months; Intervention phase-8 months; Postintervention phase-2months (Fig. 1).

## BASELINE PHASE

**Fig. 2.**
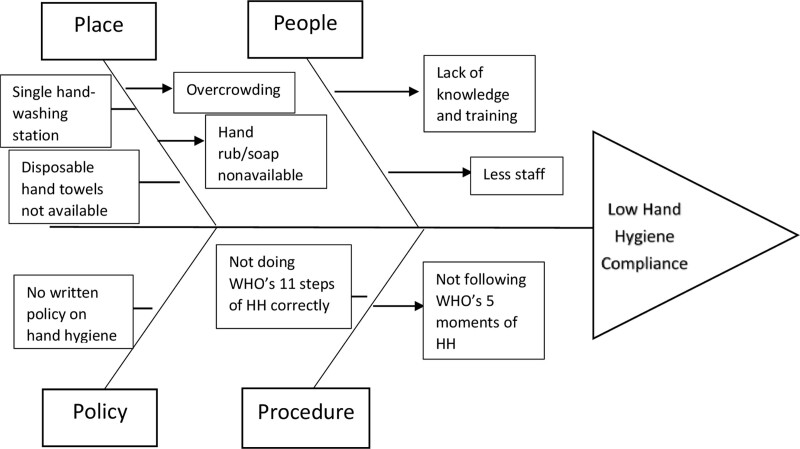
Fishbone analysis.

We constituted a multidisciplinary QI team composed of the consultant-in-charge of the SNCU, sister-in-charge, nursing staff, nursing tutor, resident doctors, and ward attendants. The QI team held multiple meetings in which the roles and responsibilities of all the members were assigned, along with training of the observers in data collection. After that, the QI team conducted and analyzed the problem’s root causes using a Fishbone diagram to identify modifiable risk factors (Fig. 2).

### Training and Data Collection

Three observers (one nursing tutor and two resident doctors) underwent training regarding the HH techniques and procedures according to World Health Organization hand hygiene 2009 guidelines. Trained observers recorded the HH compliance in the WHO HH observation tool as per standard guidelines.^8^ For data collection, we randomly selected two target SNCU patients daily by drawing lots of all admitted neonates in SNCU before the start of each day. Random selection of two admitted neonates included patients with variable clinical profiles (sick or non-sick) in the study. It made HH observation easy because it is practically difficult to observe all the neonates unobtrusively by a single observer. We divided each day into three shifts (Morning, Evening, and Night). We stopped observing HH compliance when a total number of 8 observations per patient were recorded in that shift, making 24 observations per day.

### Process Measure

We calculated the percentage of staff that performed adequate HH as a process measure. We observed the HCPs unobtrusively by hiding the HH observation tool in the patient files. All the HCPs who came in contact with the target patients were observed. We also included allied health providers (AHPs) in the project, including radiographers, ward attendants, and ward sweepers (cleaning staff). While recording HH data, we noted only the category of HCP without names in the observation form. For each observed contact with the target patient, there were 5 HH opportunities recorded separately (WHO’s “5 Moments of Hand Hygiene”). HH noncompliance to the presented opportunity (patient care activity) was defined as the inability to perform HH according to any one of the WHO’s “5 moments of hand hygiene.” For example, while handling the baby, a staff member does HH before touching but forgets to do HH after touching the baby. This opportunity will be counted as HH noncompliance. We could not record the full steps of HH technique during one shift as only one observer was available to record the data.

The observed patients’ baseline demographic and clinical characteristics included the number of indwelling devices (eg, intravenous line, umbilical arterial/venous line, arterial line, endotracheal tubes, urinary catheter, and chest drain) in a separate form. We divided the nature of contacts into low-risk and high-risk contacts based on the presumed risk of contamination or microorganism transmission (**See figure 1, Supplemental Digital Content 1**, which shows the nature of patient contacts.http://links.lww.com/PQ9/A326). We similarly continued the data collection in all three phases of the study.

### Outcome Measure

We also collated data on various types of infections (Sepsis, Pneumonia, Meningitis) and mortality rates from the ongoing data collection in SNCU to analyze the impact of HH compliance on the incidence of Healthcare-associated infection as an outcome measure. We defined HCAI in this study as any sepsis (clinical or culture-proven) occurring 48 hours after admission to the SNCU. The consultant-in-charge randomly checked the completeness and validity of the filled HH observational tool and patient baseline data every week.

### Intervention Phase

**Fig. 3.**
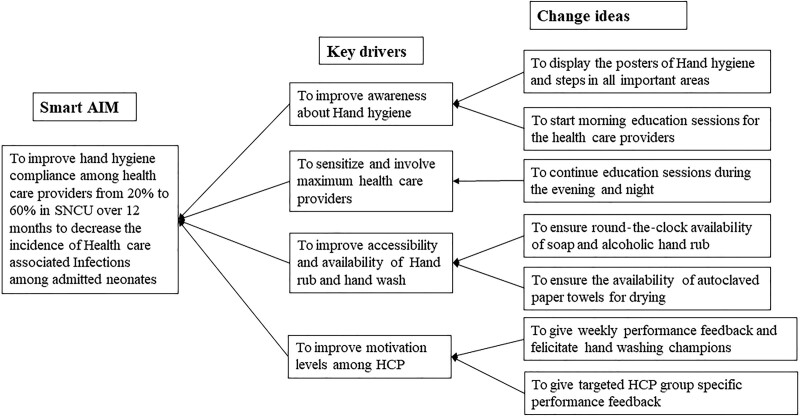
HH key driver diagram.

After root cause analysis by Fishbone diagram, the team developed a key driver diagram. They then tested a series of interventions in a typical PDSA (PLAN-DO-STUDY-ACT) cycle (Table 1 and Figure 3).

**Table 1.** Plan do Study Act (PDSA) Cycle

PDSA Cycle NO.	Timeline for Intervention (wk)	Plan	Do	Study	Act	Remarks
PDSA 1	9^th^ and 10^th^	To display the posters of Hand washing, hand rubbing steps with their indications and sensitivity sessions with the staff	Posters of Hand washing, hand rubbing steps and indications were pasted at the washbasin and other prominent places in NICU	Compliance of hand hygiene was observed	Adapted	Increase in HH compliance rate in the first week but in the second week compliance rate fell
PDSA 2	12^th^ and 13^th^	Introduction of education session every alternate day	Educational sessions on hand hygiene steps and its benefits were given	Compliance of hand hygiene increased	Adapted	Increase in HH rate in day time shifts. HCPs got to know proper steps and benefits
PDSA 3	15^th^ to 24^th^	Continued education in morning session and introduction of education sessions in the evening and night shifts	Educational sessions on hand hygiene steps and its benefits were conducted in evening and night shifts	Compliance of hand hygiene increased in evening and night shifts also	Adopted	Increase in HH rate in evening and night shifts. But, decreased availability of hand wash and hand rub in evening and night shift
PDSA 4	25^th^ to 29^th^	To increase availability of soap and hand rubs. Availability of autoclaved paper towels. Continued education 24x7	Soaps and Hand rubs were provided throughout day and night and during the weekend	Compliance of hand hygiene increased	Adopted	Increase in hand hygiene rates throughout day and night but compliance rate was around 50%.
PDSA 5	30^th^ to 35^th^	To give weekly performance feedback of whole unit	Weekly performance feedback was discussed with all HCP working in NICU by displaying compliance rates of previous week	Performance feedback was discussed without displaying personal names	Adapted	Individuals became more conscious as HH rates were discussed in meetings
PDSA 6	36^th^ to 37^th^	To felicitate the hand washing champions	Continued weekly meetings done to discuss performance feedback	Appreciation of staff by giving small gifts	Adopted	HCPs became more motivated as they were appreciated
PDSA 7	38^th^ to 40^th^	To give group specific feedback and HH education	Weekly performance feedback of specific group working in NICU	Performance feedback of specific groups were asked in meetings	Adopted	The compliance of hand hygiene of nurses and AHPs was very low. So, group-specific feedback was provided without revealing individual names.

### Postintervention Phase

This phase lasted for two months (1st May 2019 to 30th June 2019). We promoted all the interventions that led to the improvement of HH compliance in all the shifts.

### Statistical Analysis

**Fig. 4.**
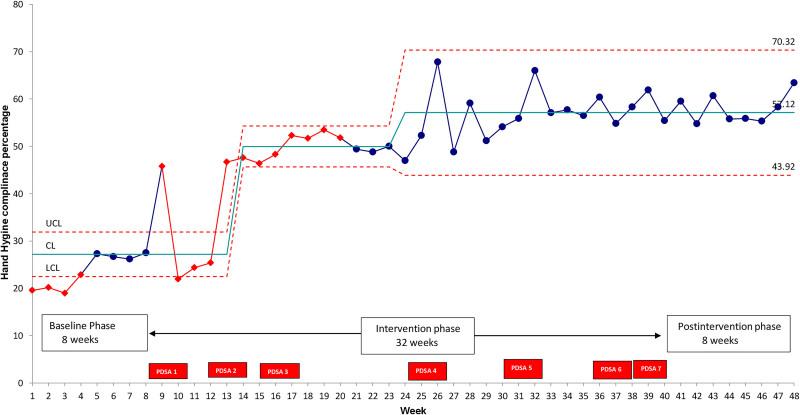
Annotated control chart for weekly HH compliance.

Data were entered in a data matrix in Microsoft Excel (Microsoft Corporation, Redman, WA) and analyzed using SPSS 20.0.0 (IBM, Corp. Armonk, N.Y.). We represented the categorical variables in frequencies and percentages and explored their association using Pearson’s chi-square test. We considered a *P* value of ≤ 0.05 as statistically significant. We depicted the primary outcome (HH compliance rate weekly) in percentages in a P chart (Fig. 4) using QI Macros Excel software (KnowWare International, Inc., Denver, CO). Data points were calculated for outcome measures every week from the start till the end of the study. We used Provost and Murray control chart rules for deciding special cause variation in our study.^9^

## RESULTS

The study lasted for one year from July 2018 to June 2019 and had three phases: Baseline (2 months), Intervention (8 months), and Postintervention (2 months). The total number of admissions in the SNCU during the study period was 939, out of which 137 were during the baseline phase, 608 were during the intervention phase, and 194 were during the postintervention phase. The number of neonates observed in the present study was 173 (baseline-29, intervention-112, postintervention-32) (Fig. 1). The number of observations was 1488, 5808, and 1464 in baseline, intervention, and postintervention phases, respectively (Fig. 1). The baseline characteristics of the observed neonates in all three phases were similar.

After introducing HH posters and sensitization of the staff in PDSA cycle 1, there was a drastic change in HH compliance in week nine, but it again came back to baseline in the 10th, 11th, and 12th weeks. So, from the 13th week onward, we started dedicated education sessions in SNCU every alternate day in the morning. After PDSA cycles 1 and 2, we found that HH compliance rates were better during the morning shift than evening and night shifts. So, in the third PDSA cycle, we started alternate day education sessions in the evening and night shifts. After that, the mean HH compliance increased to 50.0%. All these changes were adopted and practiced from week 17 to week 24; the mean HH compliance remained around 50% with no special cause variation (Fig. 4). We got feedback from our staff that there was no regular hand rub and hand wash supply, especially during the evening, night, and weekend shifts. Therefore, in the fourth PDSA cycle, we ensured round the clock availability of these items. After this intervention, there was a shift in mean HH compliance to 57.1% (Fig. 4). After that, we tried three PDSA cycles, which led to sustained improvement in mean HH compliance to 57.1% at the end of the intervention and postintervention phase (Table 1 and Figure 4).

In summary, the mean HH compliance improved from 27.2% to 57.1% in the postintervention phase (Fig. 4). The HH compliance also improved in both high-risk and low-risk contacts (Table 2). The overall HH compliance rate in doctors improved from 27.1% in the baseline phase to 60.2% and 69% in the intervention and postintervention phases, with a similar trend for both Nurses and Allied Health professionals (AHPs) (Table 2). (**See figure 2, Supplemental Digital Content 2,** which shows Run chart displaying monthly HH compliance according to the type of Health care worker (HCW) and duty shift.http://links.lww.com/PQ9/A327). The compliance rate was initially less in the evening and night shifts (25.2% and 14.9%, respectively). At the end of the intervention and postintervention phases, there was a significant increase in HH compliance in the evening and night shifts (Table 2). (**See figure 2, Supplemental Digital Content 2,** which shows Run chart displaying monthly HH compliance according to the type of Health care worker (HCW) and duty shift. http://links.lww.com/PQ9/A327.) There was a significant improvement in HH compliance for all the WHO moments except after body fluid contact. (**See table, Supplemental Digital Conntent 3**, which shows HH compliance rates according WHO’s 5 moments of hand hygiene. http://links.lww.com/PQ9/A325.) There was no difference in the HCAI and mortality between the three phases (Table 2).

**Table 2.** HH compliance rate according to type of patient contact, health care personnel, duty shift, and health care associated infections and mortality in all the three phases

Variables	Baseline N = 1488 [n/d (%)]	Intervention N = 5808 [n/d (%)]	Post intervention N = 1464 [n/d (%)]	*P*
**Type of patient contact**				
High risk	148/864 (17.1%)	1652/2990 (55.2%)	431/758 (56.8%)	<0.001
Low risk	175/624 (28%)	1245/2818 (44.1%)	390/706 (55.2%)	<0.001
Health care personnel				
Nurses	126/634 (19.9%)	968/2111 (45.8%)	262/530 (49.4%)	<0.001
Doctors	192/708 (27.1%)	1786/2964 (60.2%)	494/715 (69.1%)	<0.001
AHPs	005/146 (03.4%)	143/733 (19.5%)	065/218 (29.8%)	<0.001
Duty shift				
Morning	146/496 (29.4%)	1271/1936 (65.6%)	331/488 (67.8%)	<0.001
Evening	114/496 (22.9%)	1009/1936 (52.1%)	297/488 (60.8%)	<0.001
Night	63/496 (12.7%)	617/1936 (31.8%)	193/488 (39.5%)	<0.001
Health care associated infections and mortality				
No. admissions	137	608	194	
Healthcare Associated infection (n, %)	4 (2.9%)	29 (4.8%)	7 (3.6%)	0.93
Mortality (n, %)	1/4 (25.0%)	9/29 (31.0%)	2/7 (28.6%)	

The numerator (n) and denominators (d) are different for each type of contact as they are based on the encounters evaluated.

## DISCUSSION

This QI project improved HH compliance among the HCPs by using multiple interventions as PDSA cycles over 8 months. As the initial baseline HH compliance was very low, we kept HH compliance of 60% as our first target. In addition, we recorded a large number of HH moments (8760) during the project. As a result, we demonstrated an improvement in mean HH compliance to 57.1% from 27.2%, but there was no improvement in the rate of HCAI (Fig. 4 and Table 2).

In the study by Moghaddam et al in 2015, overall HH compliance rates rose from 30% to 70% in the postintervention phase.^10^ Similarly, Chappola et al observed increased HH compliance from 46% to 69% in the postintervention phase after training and educating health care staff.^11^We noticed that HH compliance rates were better during the morning shifts than the evening and night shifts (Table 2). (**See figure 2, Supplemental Digital Content 2,** which shows Run chart displaying monthly HH compliance according to the type of Health care worker (HCW) and duty shift. http://links.lww.com/PQ9/A327). This observation is similar to the study by Shah et al in 2015, in which they noted that the percentage of unacceptable hand washing was more prevalent at night compared with that at the daytime (17.5% versus 12.6%)^12^ After the first 4 PDSA cycles, we demonstrated improvement in HH compliance in all three shifts. However, the HH compliance during the night shift remained poor (Table 2, Figure 3). (**See figure 2, Supplemental Digital Content 2,** which shows Run chart displaying monthly HH compliance according to the type of Health care worker (HCW) and duty shift. http://links.lww.com/PQ9/A327.) In a similar 2018 study by Laskar et al. in an adult intensive care unit using a multimodal educational intervention, the HH complete adherence rate improved from 2.1% to 72.1%, 4.8% to 67.5%, and 1.9% to 68.8% in the morning, evening, and night shifts, respectively.^13^

HH compliance rates improved in the present study for both high-risk (17.1%→55.1%→56.9%) and low-risk contacts (28%→ 44.3%→55.2%) (*P* < 0.001) from the baseline to intervention and postintervention phase, respectively (Table 2). Similarly, Lam et al 2004 reported a significant improvement in HH compliance rates from 35% to 60% for high-risk procedures. However, there was a nonsignificant improvement from 43% to 49% for low-risk procedures (3). In our study, HH compliance rates improved for all healthcare staff (Table 2). Similar results were found by Moghaddam et al in 2015, where compliance rates rose from 33.5% to 80.6% for doctors, 29.4% to 66.2% for nurses, and 24.2% to 56.4% for AHPs from pre-intervention to postintervention phase.^10^ We did not target and involved AHPs for HH education in the early part of the intervention phase, which may be the reason for low overall HH compliance among AHPs (Table 2). We sequentially introduced the practice of displaying weekly unit performance, felicitation of handwashing champions, and group-specific performance in the 5th, 6th, and 7th PDSA cycle, respectively, which led to increased awareness, especially among AHPs (Fig. 3). (**See figure 2, Supplemental Digital Content 2**, which shows Run chart displaying monthly HH compliance according to the type of Health care worker (HCW) and duty shift. http://links.lww.com/PQ9/A327.)

There was no significant change in HCAI rates over the three study phases in the present study. Many other factors like overcrowding and a lower nurse-to-patient ratio also increased HCAI (Table 2). Our results are in concordance with the study by Mukerji et al., where there was no significant improvement in HCAI rates.^14^ In contrast, Moghaddam et al. found that HCAI rates decreased significantly from 5.4% to 1.7% in the postintervention phase.^10^ In our study, there was no significant change in mortality rates (Table 2). This result contrasts with Moghhaddam et al, where mortality rates decreased from 14.5% to 8.9% in the postintervention phase.^10^

Our study’s strengths were that we recorded many HH moments during all three shifts of the day. In addition, we randomly selected patients for observation by a process that removed selection bias and involved patients with variable clinical profiles. Thus, this report is one of the first studies in India that collated data on HH compliance according to patient contact, health care personnel, and duty shift in the SNCU setting. However, as is the case with all the studies, our study also had a few limitations. For instance, we could not record the full HH technique consistently during the study period as during one shift, as only one observer was there to record the data. So, we did not have consistent data on the proportion of individual HH steps done correctly. Also, we did not perform interrater reliability testing following the training of the observers, which may affect the quality of data collection. Moreover, we could not collect data on balancing measures like an increase in the incidence of dryness and rash on the hands of Health care providers due to increased usage of alcohol rub. This issue can also be one reason for poor HH compliance, especially among nurses and AHPs.

The HH compliance rates improved significantly but not to the desired extent due to comparatively lower compliance rates among nurses and AHP. In the future, we are also planning to increase our workforce and introduce an orientation and training program for all the newly recruited staff. Although the hospital administration has been upraised about our study findings, very soon, a hospital-level HH policy will be formulated and implemented in other units using the QI approach. One of the positive effects of this project was that our team members are now more enthusiastic to carry on this critical intervention to the next level. So, we can proudly say that this has cultivated the culture of QI and teamwork in our unit. This project again reiterates that interventions must be more specific and regular to modify human behavior, such as HH habits. System or administrative changes like round-the-clock availability of hand wash/hand rub, provision of an adequate number of handwashing stations, less overcrowding, improving nurse–patient ratio, and regular HH audits are essential for sustainability. Our study’s results suggest that the health care staff and doctors in our SNCU were amenable to change with an appropriate educational program. With system changes suggested above, a health facility can achieve HH compliance close to 100%.

## CONCLUDING SUMMARY

A multimodal QI study improved HH compliance in our SNCU. This study’s methods and results can guide the tertiary care SNCUs in the public health sector to improve HH compliance in their setting. The future QI studies on HH compliance should focus on administrative and infrastructural issues like the number of handwashing stations in SNCU, rational admission policy, and provision of step-down units to decrease overcrowding, staff number, and disproportionate staff posting in the three shifts along with training and education of health care staff.

## DISCLOSURE

The authors have no financial interest to declare in relation to the content of this article.

## ACKNOWLEDGMENT

We thank the Hospital administration for supporting us in every possible way. We also thank the health care staff working in SNCU; without them, this study would not have been possible.

## Supplementary Material


