# Supplementary material for: Quality Improvement Initiative to Improve Hand Hygiene Compliance in Indian Special Newborn Care Unit
Source: Pediatr Qual Saf. 2021 Dec 15;6(6):e492. doi: 10.1097/pq9.0000000000000492 (PMC8678003; doi:10.1097/pq9.0000000000000492)
Supplement: Supplementary file 1 [file pqs-6-e492-s001.pdf]

Panel 1: Nature of Patient Contacts

High-risk contacts

- Invasive procedures like inserting intravenous catheter and taking blood sample
- Handling mucous membrane secretions, open wounds and body fluids
- Administering intravenous and intramuscular medication
- Changing Intravenous fluids
- Insertion of nasogastric feeding tube
- Caring of Peripheral Inserted Central (PICC) line
- Endotracheal intubation or suction
- Prolonged contacts like changing position in an intubated neonate, chest physiotherapy

Low-risk contacts

- Physical examination and vital signs assessment
- Giving oral medication
- Cup/Tube feeding
- Nesting/ Swaddling/ Assisting in Kangaroo mother care position
- Attachment of pulse oximeter probe
