# Supplementary material for: Quality Improvement Initiative to Improve Hand Hygiene Compliance in Indian Special Newborn Care Unit
Source: Pediatr Qual Saf. 2021 Dec 15;6(6):e492. doi: 10.1097/pq9.0000000000000492 (PMC8678003; doi:10.1097/pq9.0000000000000492)

Supplementary Figure 2: Run chart showing monthly HH compliance according to type of Health care worker (HCW) and duty shift

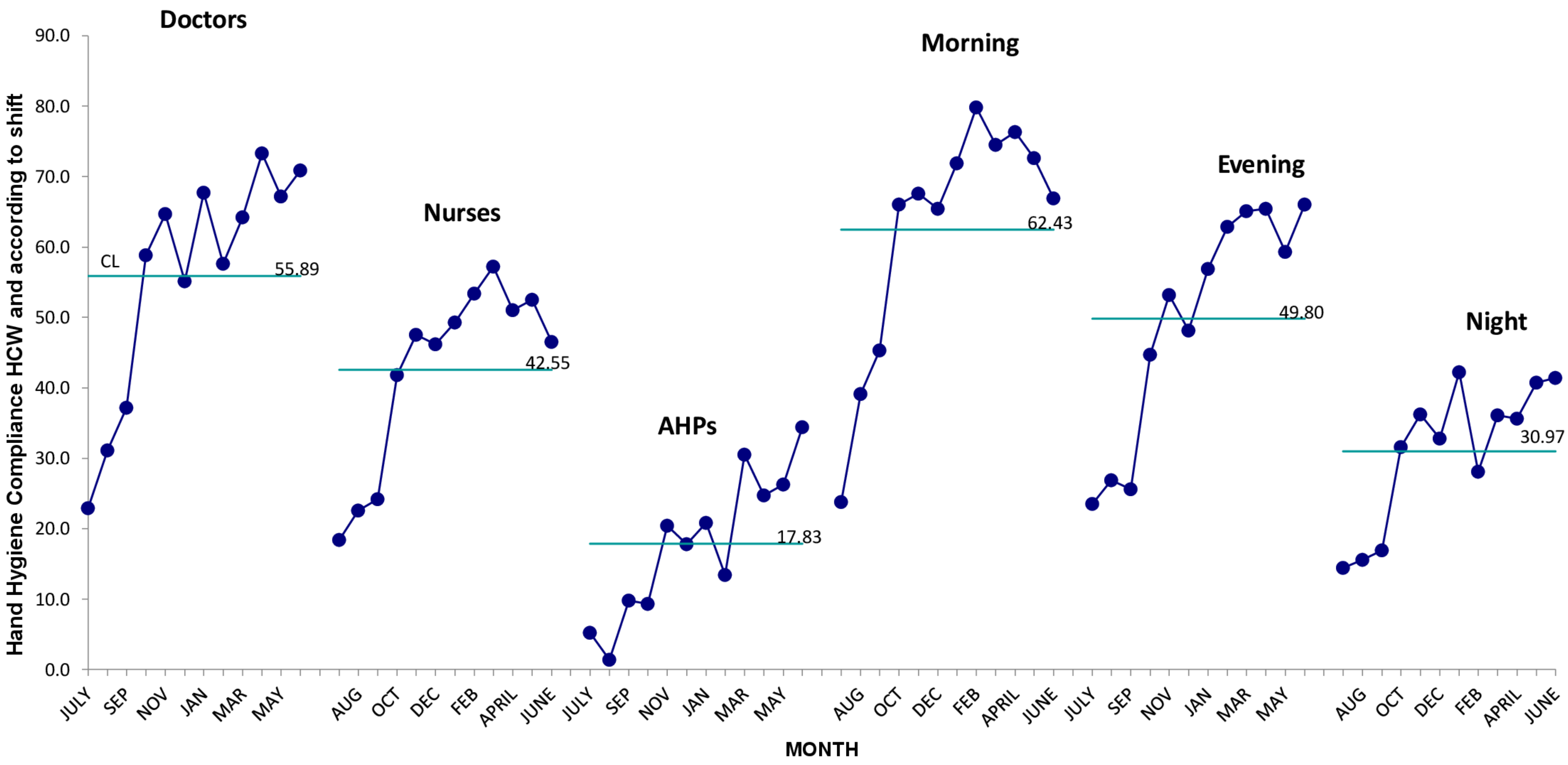

Supplement: Supplementary file 2 [file pqs-6-e492-s002.pdf]
