# Supplementary material for: Quality Improvement Initiative to Improve Hand Hygiene Compliance in Indian Special Newborn Care Unit
Source: Pediatr Qual Saf. 2021 Dec 15;6(6):e492. doi: 10.1097/pq9.0000000000000492 (PMC8678003; doi:10.1097/pq9.0000000000000492)
Supplement: Supplementary file 3 [file pqs-6-e492-s003.pdf]

Supplementary table 1: HH compliance rates according WHO's 5 moments of Hand Hygiene

Phases of study				
	Baseline	Intervention	Post-intervention	p Value
Total HH moments observed	1488	5808	1464	
• Before patient contact (n)	412	1504	408	
• Before aseptic procedure (n)	496	1836	491	
• After body fluid contact (n)	098	997	092	
• After patient contact (n)	398	1324	356	
• After patient surroundings (n)	084	147	117	
**Compliance rates**				
• Before patient contact (n,%)	74/412 (17.9%)	725/1504 (48.2%)	202/408 (49.5%)	<0.00001
• Before aseptic procedure(n,%)	103/496 (20.7%)	929/1836 (50.5%)	288/491 (58.6%)	<0.00001
• After body fluid contact (n,%)	41/98 (41.8%)	482/997 (48.3%)	49/92 (53.2%)	0.134 NS
• After patient contact (n,%)	91/398 (22.8%)	689/1324 (52.0%)	223/356 (62.6%)	<0.00001
• After patient surroundings(n,%)	14/84 (16.6%)	72/147 (48.9%)	59/117 (50.4%)	<0.00001

Note: The denominators are different for each of the five moments as they are based on the encounters evaluated

p<0.001-highly significant, *Chi square test
